# Absolute numbers of regulatory T cells and neutrophils in corticosteroid-free patients are predictive for response to bevacizumab in recurrent glioblastoma patients

**DOI:** 10.1007/s00262-019-02317-9

**Published:** 2019-03-04

**Authors:** Véronique Quillien, Antoine F. Carpentier, Alain Gey, Tony Avril, Eric Tartour, Floraly Sejalon, Boris Campillo-Gimenez, Elodie Vauleon

**Affiliations:** 1 Centre Eugène Marquis, rue bataille Flandres Dunkerque, CS44229, 35042 Rennes, France; 2 Chemistry Oncogenesis Stress Signaling Laboratory, INSERM U1242, Université de Rennes 1, Rennes, France; 3 Hôpital Saint-Louis, AP-HP, Paris, France; 4 Hôpital Européen Georges Pompidou, Service d’Immunologie Biologique, Paris, France; 5 INSERM U970, Universite Paris Descartes, Paris, France; 6 INSERM U1099, Université de Rennes 1, Rennes, France

**Keywords:** Glioblastoma, Bevacizumab, Neutrophils, Regulatory T cells, Biomarker

## Abstract

Bevacizumab (Bv) remains frequently prescribed in glioblastoma (GBM) patients, especially at recurrence. We conducted a prospective clinical trial with 29 recurrent GBM patients treated with Bv alone with a longitudinal follow-up of different circulating immune cells [complete blood count, myeloid-derived suppressor cells (MDSCs), classical, intermediate, non-classical and Tie2 monocytes, VEGFR1+ and regulatory T cells (Treg)]. We observed a significant increase for leucocytes, neutrophils, eosinophils and classical monocytes and a decrease for the fraction of Treg during the treatment. The best prognostic values for survival under Bv were obtained for basal neutrophils and Treg. Counts below 3.9 G/L for neutrophils and above 0.011 G/L for Treg were associated with an overall survival of 17.5 and 19.9 months, respectively, as compared with 5.4 and 5.6 months, respectively, for counts above and below these cutoffs (*p* = 0.004 and *p* < 0.001). No prognostic impact was observed for neutrophils in a retrospective cohort of 26 patients treated with nitrosoureas alone. In another retrospective validation cohort of 61 GBM patients treated at recurrence with a Bv-containing regimen, an interaction was observed between neutrophils and corticosteroid intake. The predictive value of neutrophils on survival under Bv was lost in patients treated with corticosteroids, when steroid-free patients with a low neutrophil count had a particularly long median survival of 3.4 years. These two simply accessible criteria (basal neutrophils and steroid intake) could be used to reserve this relatively costly treatment for patients likely to be the most responsive to Bv and prevent unnecessary side effects in others.

**Electronic supplementary material:**

The online version of this article (10.1007/s00262-019-02317-9) contains supplementary material, which is available to authorised users.

## Introduction

The standard of care for glioblastoma (GBM) patients at recurrence is not well defined. Bevacizumab (Bv) is often prescribed in various countries because two phase II studies initially showed that approximately 30% of patients responded to treatment, with improved progression-free survival (PFS) [1, 2]. This led to the randomised controlled phase II BELOB trial, in which the rate of overall survival at 9 and 12 months was higher with the combination of Bv and lomustine than with either agent alone [3]. Yet, in the large EORTC 26101 phase 3 trial, adding Bv to lomustine failed to increase survival over lomustine alone, although the PFS was longer (4.2 months) in the combination group than in the monotherapy group (1.5 months) [4]. Despite these mixed results, Bv remains a therapeutic option for GBM patients. This is explained, in part, by the fact that this treatment can have corticosteroid-sparing effects, but also because a proportion of patients show long-term response to Bv. Several attempts have been made to identify this subset of patients to reserve this relatively costly treatment to responsive patients and prevent unnecessary side effects in others.

Vascular endothelial growth factor (VEGF), the molecular target of Bv, was previously seen as an obvious predictive marker of response to Bv. However, most studies in gliomas as well as in other pathologies have failed to show a correlation between a high level of VEGF detected in the circulation or in the tumour and outcomes of patients [5, 6]. Other studies aimed at identifying a link between the molecular profile of the tumour and the response to Bv-containing regimen. Patients from the BELOB trial with an initial tumour assigned to the specific molecular subtype of glioma, IGS-18, or classical GBMs, showed more benefit from Bv/CCNU treatment. This benefit was not found for patients treated with Bv only [7]. In a smaller retrospective cohort of patients treated at recurrence with a combination of Bv and irinotecan, patients with initial tumours assigned to IGS-18 or classical GBMs had, on the contrary, worse outcomes than those assigned to IGS-22/23 [8]. A microRNA profile of tumour tissue has been described to have a predictive potential in GBM patients treated with Bv [9]. Differential expression of single genes, as angiotensinogen and HLA class II, has also been reported to predict Bv response in recurrent patients treated with the same combined treatment [10]. A major limitation of these studies is the use of primary tumour tissue for classification/analysis, whereas the treatment targets the recurrent tumour. As the recurrent tumour is rarely available for GBM patients, looking for blood biomarkers may be a better alternative.

In this study, we analysed different blood circulating immune cells at the time of relapse. Infiltrating immune cells are recognised to play a crucial role in regulating the formation and the remodelling of tumoural blood vessels and can regulate responsiveness and resistance to antiangiogenic therapies [11]. The great majority of these cells are bone marrow-derived cells recruited to the tumour via the blood. Our hypothesis was that the inhibition of angiogenesis at the tumour level, following the blockade of VEGF by Bv, would lead to changes in the recruitment of immune cells, inducing variations in their peripheral levels. A first goal was to conduct a longitudinal follow-up of different subsets of immune cells in the peripheral blood of recurrent GBM patients during their course of Bv treatment. A second goal was to analyse the potential prognostic value of enumeration of these cells. To achieve this goal, we conducted a prospective clinical trial, in which patients were treated at relapse by Bv alone. Some of the results were further validated in two independent retrospective cohorts of GBM patients.

## Materials and methods

### Patients and study design

#### Initial prospective cohort

Recurrent GBM patients for whom a Bv treatment was planned as part of their care were enrolled in this bicentric prospective study. The primary goal of the study was to perform a longitudinal follow-up of different circulating immune cells and to highlight those whose rates varied significantly during the course of treatment. The secondary goal was to search for a link between immune cell levels and overall patient survival. Patients were above the age of 18 years, had a proven progressive disease occurring at least 3 months after radiotherapy and 4 weeks after chemotherapy (if appropriate) and were eligible for a Bv treatment. After inclusion, they were treated with Bv 10 mg/kg i.v. every 2 weeks until progression.

#### Validation cohorts

GBM patients treated at first recurrence with Bv alone or in association with camptothecin or nitrosoureas (cohort 1) or nitrosoureas alone (cohort 2) and for whom a complete blood count was available before treatment were selected.

### Analysis of blood samples

Fifteen millilitres of peripheral venous blood was collected in K2 EDTA tubes before initiation of Bv and then before the third, fifth and seventh cycles of treatment. The flowchart of the study design is illustrated in Sup. Figure 1. For each point, a complete blood cell count (CBC) was performed on a Beckman Coulter haematology analyser. A six-colour flow cytometric analysis on fresh blood was also performed using a BD FACSCanto II cytometer (Rennes) or a Beckman cytometer (Paris). Flow cytometry analyses were performed using FACSDiva (BD Biosciences) and Kaluza (Beckman Coulter) software. The gating strategies are described in detail in Figure S2. Negative controls were included for defining accurate gating. Preliminary tests made it possible to ensure that identical results were obtained at the two sites carrying out the analysis. G-MDSCs (granulocytic myeloid-derived suppressor cells) were identified as CD33^+^/CD203^−^/CD14^−^/CD16^−/low^/HLA-DR^−^. Classical, intermediate and non-classical monocytes were identified as CD14^high^/CD16^−^, CD14^high^/CD16^+^ and CD14^low^/CD16^+^, respectively. Tie 2 expressing monocytes (TEM) were identified as CD14^+^/Tie2^+^, M-MDSCs (monocytic myeloid-derived suppressor cells) as CD14^+^/HLA-DR^−/neg^ and Treg as CD3^+^/CD4^+^/CD25^high^/FoxP3^+^. VEGFR1 expression was analysed on CD14^+^ cells and total leucocytes (cytometric analysis based on cell size and granularity). For each population, an absolute number was calculated as follows: (total white blood cell count × percent of the population of interest among leucocytes)/100. For Treg, the percentage of Treg among CD3^+^ T cells and among CD3^+^/CD4^+^ T cells was also determined. For a detailed description of the antibodies, the methods used for labelling and the gating strategies, see Sup. Figure 2.

For the validation cohorts, data were extracted from CBC done as part of the standard monitoring of the patients.

### Statistical analysis

Data were described using median [minimum–maximum] values concerning quantitative variables and frequencies *n* (%) for categorical variables. Biological counts were reported in the form of cell counts and percent out of cell populations of interest (e.g. % of Treg among CD3^+^ T cells). Box plots were presented to draw evolution of biological counts at each sample time: before C1 (the first cycle noted C1), C3, C5 and C7. Evolution over time of the cell populations was tested through one-way ANOVA with repeated measures. Further pairwise comparisons from the baseline value (C1) were conducted with Wilcoxon matched pairs signed-ranks tests. No adjustment for alpha risk inflation was performed, but figure presentation allows reproducing such kind of reasoning by distinguishing *p* < 0.5, 0.01 and 0.001.

Overall survival was defined as the time between inclusion in the study and death. Survival rates were estimated by the Kaplan–Meier method and survival curves compared by log-rank tests. Each explanatory factor was dichotomised by the median value at C1 and the median value of the ratio to baseline after two cycles (C3). Survival medians and 95% confidence intervals are presented as M [95% IC lower bound–95% IC upper bound].

Results from the prospective cohort were then confirmed on two different historical cohorts of patients having received Bv (*n* = 61) or not (*n* = 27) at recurrence. Steroid intake at recurrence, neutrophil counts (> median) and interactions between biological parameters and steroid intake were introduced in a multivariable Cox model.

Analysis was conducted with the R Core Team software (2014): a language and environment for statistical computing. R foundation for statistical computing, Vienna, Austria (http://www.R-project.org/).

## Results

### Studied populations

Twenty-nine patients (21 in Rennes and 8 in Paris) were enrolled in the prospective study from September 2012 through December 2014. The median overall survival was 8.5 months [6.8–18.4]. To constitute the validation cohort 1, all the GBM patients having received Bv at recurrence, not having participated in the prospective protocol and for whom a total blood count was available within 10 days before the start of treatment were extracted from the local database. Sixty-one patients treated between December 2008 and May 2017 were selected. Among this cohort, 60% of patients received Bv alone as a second-line treatment and 56% received a combined treatment (Bv and chemotherapy) as a second or third line. In the prospective cohort, half of the patients received a combined treatment (Bv and chemotherapy) after failure of Bv as monotherapy, which makes these two cohorts relatively similar. The validation cohort 2 included 27 patients treated by nitrosourea alone between the dates of April 2009 and October 2015. The medians for overall survival were 8 months [6–11] and 9 months [5–17] for cohorts 1 and 2, respectively. Clinical patient characteristics are summarised in Table 1.

**Table 1:** Baseline characteristics of the patients

	Prospective cohort (*n* = 29)	Validation cohort 1 Bv+/− CT (*n* = 61)	Validation cohort 2 CT only (*n* = 27)
Age (years)			
Median	59.1	56.9	59.9
Range	(32–76)	(31–79)	(37–81)
Use of glucocorticoids [*n* (%)]			
No	13 (45%)	18 (30%)	13 (48%)
Yes	16 (55%)	43 (70%)	14 (52%)
Median survival (months)			
OS	8.5	8.0	9.0

*Bv* bevacizumab, *CT* chemotherapy, *PFS* progression-free survival, *OS* overall survival

### Levels of different immune cells vary significantly during Bv treatment

**Fig. 1:**
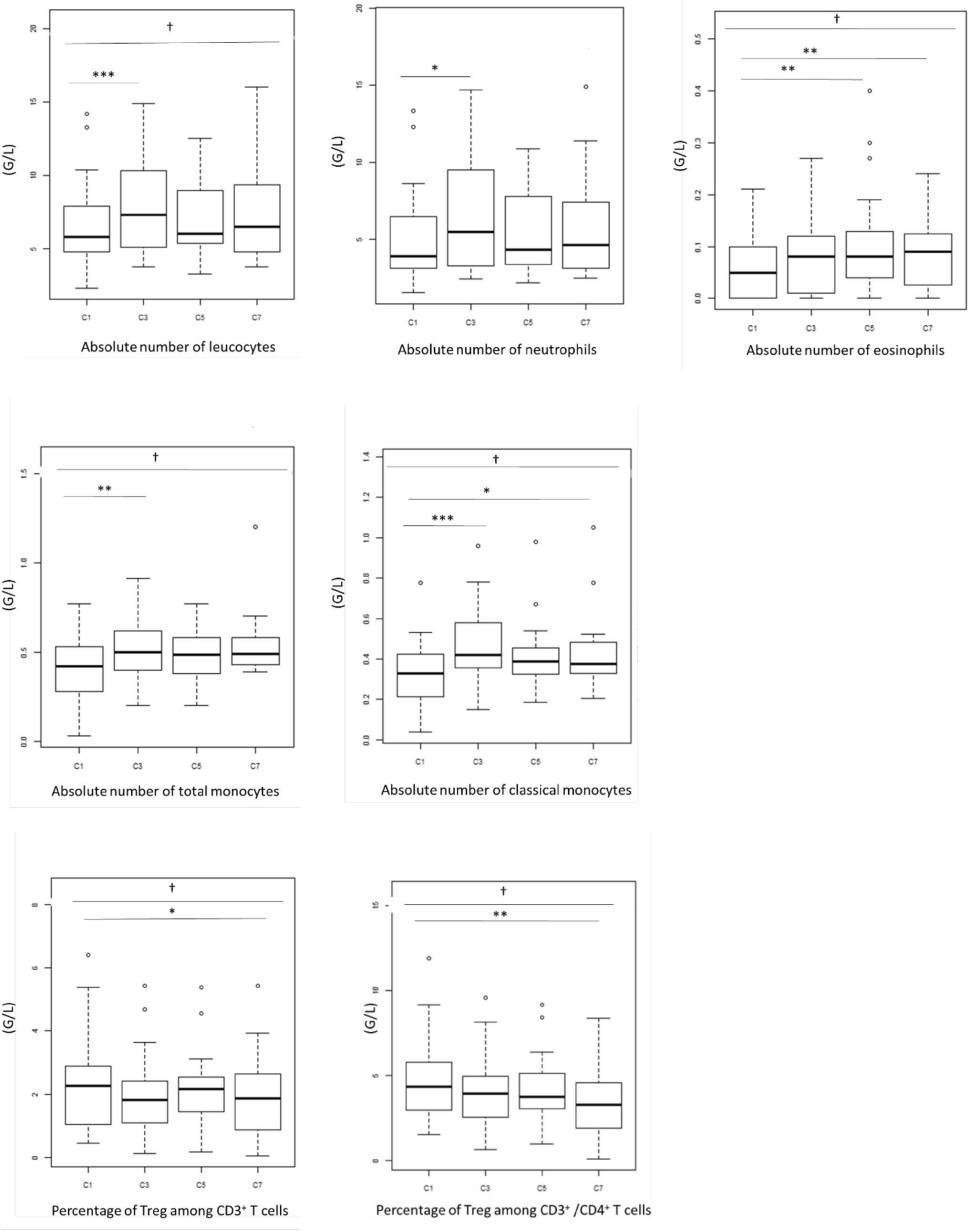
Immune cells with significant variations during bevacizumab treatment. A complete blood cell count (CBC) and a cytometric analysis were assessed at baseline (C1) and before the third (C3), the fifth (C5) and the seventh (C7) cycles of treatment. Leucocyte, neutrophil, eosinophil, total monocyte and classical monocyte counts increased, whereas percentage of Treg among CD3^+^ and CD3^+^/CD4^+^ T cells decreased during treatment. The bottom of the box-and-whisker plot graph shows the 25th percentile of the variable, the line within the box indicates the median, and the top of the box shows the 75th percentile. Ends of the whiskers are at 25th percentile − (1.5 × interquartile range) and 75th percentile + (1.5 × interquartile range). Outliers are indicated as small circles. † indicates *p* values for global change during treatment (*p* ≤ 0.05^†^, *p* ≤ 0.01^††^, one-way repeated ANOVA). * indicates *p* values for changes between baseline and the subsequent cycles (*p* ≤ 0.05*, *p* ≤ 0.01**, *p* ≤ 0.001***, Wilcoxon test)

As shown in Fig. 1, significant variations were recorded for total leucocytes during treatment, with a notable increase between the samples taken before treatment (5.8 G/L [2.3–14.2]) and the one taken before the third cycle (7.3 G/L [3.8–14.9]) (*p* = 0.001). This increase was mainly due to the contingent of myeloid cells. Indeed, a similar profile was observed with neutrophils (3.9 G/L [1.54–13.35]) at C1, (5.5 G/L [2.45–14.71]) and C3 (*p* = 0.022) and with monocytes (0.42 G/L [0.03–0.77]) at C1 (0.5 G/L [0.20–0.91]) and C3 (*p* = 0.005), whereas lymphocyte levels remained stable during the treatment. Among the monocytes, the increase was in the large majority population of classical monocytes. The absolute CD3^+^CD4^+^CD25^+^FOXP3^+^ Treg count did not change during the course of treatment, whereas a decrease in the percentage of Treg among CD3^+^ T cells and CD3^+^/CD4^+^T cells was observed with 2.26% and 4.325% at baseline and 1.86% and 3.265% at C7, respectively (*p* = 0.016 and 0.006). No significant variation was observed for the other analysed immune cells when considered as a percentage among leucocytes (for monocytes and MDSCs) or among CD14^+^ cells (for monocytes).

### Basal neutrophil and Treg counts have the best prognostic values for overall survival

**Fig. 2:**
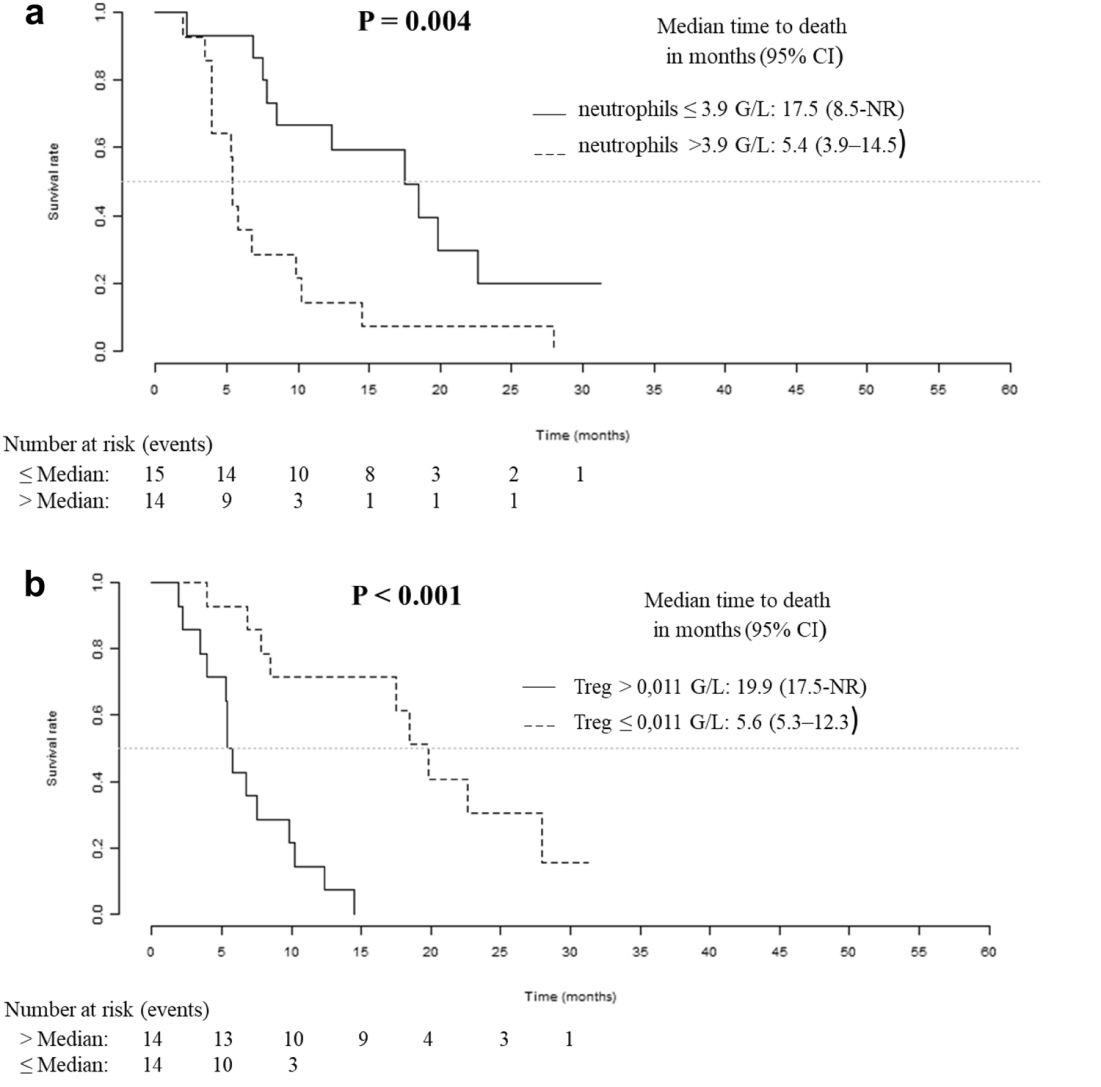
Overall survival for the initial cohort of patients. Kaplan–Meier analysis of overall survival according to basal neutrophil (**a**) and Treg (**b**) counts

For each cell population, the prognostic impact of the baseline level and the ratio to baseline level after two cycles of treatment were tested. As shown in Table 2, a high baseline level (above the median) of total leucocytes, neutrophils and platelets had a significant pejorative impact on overall survival. Conversely, a high baseline level (above the median) of intermediate monocytes, non-classical monocytes and Treg had a positive impact on overall survival. The two best predictors of survival in our series of patients treated with Bv were the basal levels of neutrophils and Treg. No prognostic impact was found for the ratio to baseline level after two cycles of treatment. Figure 2 presents the plots of Kaplan–Meier survival curves showing the overall survival of patients dichotomised according to the median values. Patients with an absolute neutrophil count above 3.9 G/L had a median overall survival of 5.4 months [3.9–14.5], whereas patients with neutrophils below or equal to 3.9 G/L had a median overall survival of 17.5 months [8.5-NR] (*p* = 0.004). Patients with an absolute Treg count above 0.011 G/L had a median overall survival of 19.9 months [17.5-NR], whereas others had a median overall survival of 5.6 months [5.3–12.3] (*p* < 0.001).

**Table 2:** Cell populations with a prognostic impact on overall survival

Cell population	Baseline level	
	Cutoff (G/L)	*p*
**Total leucocytes (CBC)**	**5.8**	**0.01**
**Neutrophils (CBC)**	**3.9**	**0.004**
**Platelets (CBC)**	**189**	**0.022**
Intermediate monocytes (FCM)	0.023	0.026
Non-classical monocytes (FCM)	0.013	0.027
Treg (FCM)	0.011	< 0.001

Patients were dichotomised according to the median value of the different cell populations. Comparison of survival curves was done using the log rank test. Populations with a negative impact on survival appear on bold

*CBC* complete blood cell count, *FCM* flow cytometry

Among clinical variables available, age was not a prognostic variable, when baseline corticosteroid treatment was associated with reduced survival in univariate analysis (*p* < 0.001). Unlike neutrophils, Treg had a prognostic impact regardless of steroid in bivariate analysis (Table 3).

**Table 3:** Univariate and bivariate survival analysis

	Univariate analysis		Bivariate analysis	
	HR (95% CI)	*p* value	HR (95% CI)	*p* value
Prospective cohort (Bv)				
Corticosteroid use (yes)	4.8 [1.90; 12.12]	< 0.001	3.7 [1.31; 10.22]	0.013
Neutrophil count > 3.9 G/L	3.31 [1.43; 7.65]	0.005	1.96 [0.76; 5.05]	0.164
Prospective cohort (Bv)				
Corticosteroid use (yes)	4.8 [1.90; 12.12]	< 0.001	2.1 [0.65; 6.96]	0.212
Treg count > 0.011 G/L	0.13 [0.04; 0.40]	< 0.001	0.21 [0.05; 0.83]	0.026
Validation cohort 1 (Bv+/− CT)				
Corticosteroid use (yes)	3.2 [1.58; 6.56]	0.001	7.2 [1.84; 27.94]	0.005
Neutrophil count > 3.9 G/L	3.2 [1.55; 6.80]	0.002	5.9 [1.63; 21.61]	0.007
Interaction	–	–	0.16 [0.03; 0.78]	0.024

*HR* hazard ratio, *CI* confidence interval

### Neutrophil count has a high positive predictive value of response to Bv, only in steroid-free patients

**Fig. 3:**
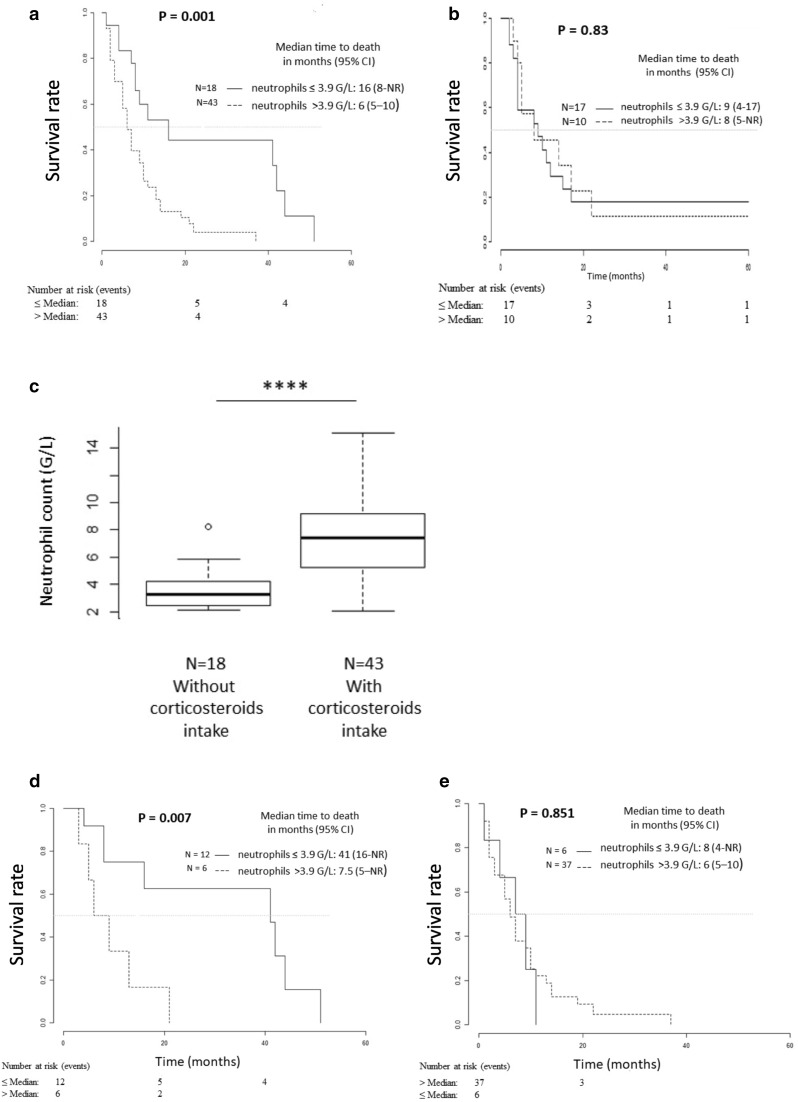
Overall survival for the validation cohorts and impact of corticosteroid intake. Kaplan–Meier analysis of overall survival according to basal neutrophil in a series of 61 glioblastoma patients treated at recurrence with bevacizumab alone or with chemotherapy (**a**) and in a series of 26 glioblastoma patients treated at recurrence with nitrosoureas (**b**). **c** Impact of corticosteroid intake on neutrophil count in the series of 61 patients. The bottom of the box-and-whisker plot graph shows the 25th percentile of the variable, the line within the box indicates the median and the top of the box shows the 75th percentile. The ends of the whiskers are at 25th percentile − (1.5 × interquartile range) and 75th percentile + (1.5 × interquartile range). Outliers are indicated by small circles. **** indicates the *p* value of the neutrophil counts of patients taking corticosteroids versus those not taking corticosteroids (*p* = 1.3 × 10^−7^, calculated by *t* test). Kaplan–Meier analysis of overall survival according to basal neutrophil count in patients without corticosteroids (**c**) or with corticosteroids (**d**) and receiving a bevacizumab containing regimen

Treg results could not be validated in retrospective data as it requires flow cytometry that is not routinely performed. On the contrary, this could be done in two independent cohorts for neutrophil counts, using the previously determined cutoff 3.9 G/L. In the cohort of 61 patients treated at recurrence with Bv with or without chemotherapy, the results were similar to those obtained during the prospective trial. Patients with an absolute neutrophil count above 3.9 G/L had a median overall survival of 6 months [5–10], whereas patients below 3.9 G/L had a median overall survival of 16 months [8-NR] (*p* < 0.001) (Fig. 3a). On the other hand, Impact on overall survival of neutrophils was not found in the cohort of 26 patients treated at recurrence with nitrosourea alone (Fig. 3b). This indicates that neutrophil count is predictive of response to Bv in GBM patients at recurrence.

As observed in the prospective cohort, age was not a prognostic variable (data not shown), when baseline corticosteroid treatment was associated with reduced survival in univariate analysis (*p* = 0.001) (Table 3). On the other hand, both corticosteroids and neutrophils remained significant in bivariate analysis, with a positive interaction between these two variables (*p* = 0.024) (Table 3).

The neutrophil count was 3.3 G/L [2.1–8.2] in the population that did not take corticosteroids at the beginning of Bv treatment compared to 7.4 G/L [2.1–15.1] for the patients on corticosteroids (*p* = 1.4 × 10^−5^, Wilcoxon test) (Fig. 3c). It should be noted that among the 18 patients without corticosteroids at baseline treatment, 11 had received no corticosteroids during their previous postoperative treatment and could therefore be considered ‘glucocorticoid-naïve’. After stratification of patients according to corticosteroid treatment, predictive value of neutrophil count remained significant only in the population without corticosteroid intake at recurrence (*n* = 18), resulting in a better overall survival for patients with low neutrophils counts of 41 months [16-NR], compared to 7.5 months [5-NR] for patients with high neutrophil counts (*p* = 0.007) (Fig. 3d, e).

## Discussion

In this study, we were able to show that different populations of circulating cells vary significantly in GBM patients treated at recurrence with Bv, especially with an increase in the absolute number of different subsets of myeloid cells that occurs after the first two cycles of treatment. The only significant observed reduction was for the percentage of Treg among CD3^+^ or CD3^+^/CD4^+^ T cells. A similar reduction in blood Treg percentage, linked to a decrease in their proliferation, has been reported for metastatic colorectal cancer after two cycles of Bv plus chemotherapy [12]. In our cohort of patients, this decrease had no impact on patients’ behaviour, whereas it correlates with a better overall survival in metastatic renal cancer patients treated with sunitinib, a multitargeted receptor tyrosine kinase inhibitor (TKI) including VEGFR types 1 and 2 [13]. In GBM patients treated at recurrence with axitinib, a selective inhibitor of VEGFR-1, 2 and 3, a significant increase of the percentage of Treg within CD4^+^ T cell was observed after 6 weeks of treatment in the case of progressive disease [14].

The best correlations with survival, in our prospective cohort of patients, were found for factors analysed before treatment, not for decrease or increase of a particular subset of immune cells. It seems therefore that the pre-existing state of the patient conditions the response to treatment, rather than the specific activity of Bv on the different immune cells analysed. More than half of the patients were receiving corticosteroids before the introduction of Bv at recurrence, which indicates that they had neurologic symptoms. These patients had a shorter survival, which could be linked to a poorer performance status and/or a greater tumour volume. One cannot also exclude a direct deleterious effect of corticosteroids. Retrospective clinical analyses have indeed identified corticosteroid use during first-line radiotherapy as an independent indicator of shorter survival in three independent cohorts of GBM patients [15].

Patients receiving corticoids had higher neutrophil counts than steroid-independent patients. This is a well-known effect of corticosteroid intake, due in particular to their demargination from the endovascular lining of the blood vessels [16], which can explain why the prognostic value of neutrophil count was lost for these patients. On the other hand, the basal neutrophil count refined the prognosis among patients without corticosteroids, with the identification of patients with a particularly long median survival of 3.4 years from the beginning of Bv treatment.

Neutrophils are the most abundant white blood cells in humans. It has now been well established that these cells are not only terminally differentiated involved in infection, but can also be found in different stages of maturation and activation into the blood from which they can extravasate into tissues. Especially, a population of circulating CD49d^+^/VEGFR1^high^/CXCR4^high^ neutrophils that stimulates angiogenesis has been described in humans and mice. These neutrophils represent a small population in healthy humans and are recruited to tissue by VEGF-A [17]. Glioma models suggest that increased recruitment of neutrophils during anti-VEGF therapy promotes glioma progression with a mesenchymal switch and may promote tumour resistance [18]. Neutrophils may also be involved in immunosuppression. In GBM patients, an expanded population of circulating degranulated neutrophils characterised by a decrease in density and an increase of arginase release inducing a peripheral immunosuppression has been described [19]. These neutrophils could correspond to the recently described immunosuppressive neutrophils, characterised by the expression of CD10+ that can inhibit proliferation and interferon gamma production by T cells via a CD18-mediated contact-dependent arginase 1 release [20]. It would certainly be interesting to distinguish these different neutrophil subtypes in GBM patients using these new markers.

MDSCs are also myeloid cells frequently described as involved in GBM immunosuppression. These cells are a heterogeneous population of cells at different stages of differentiation. At least two distinct MDSC subpopulations have been identified in humans: the granulocytic subset (G-MDSCs) and the monocytic subset (M-MDSCs). An increased number of both types of circulating MDSCs has been reported in the PBMC (peripheral blood mononuclear cells) of GBM patients compared to normal donors [21, 22]. The number of MDSCs remains, however, relatively low compared to neutrophils, with an average of 40 neutrophils for one MDSC in our study. Furthermore, we show here that the number of MDSCs does not vary significantly during Bv treatment and does not correlate with patient survival.

A pre-treatment neutrophil–lymphocyte ratio (NLR) has been shown to be prognostic in several solid tumours. For newly diagnosed GBM patients treated according to the Stupp protocol, no less than four studies have reported that a high ratio (4.1–7.5, depending on the study) before treatment is correlated with a shorter survival [23–26]. This ratio is regarded as a marker of systemic cancer-associated inflammation. In our prospective cohort, we observed that a median basal NLR of 5.2 and patients with a value above the median had no significant shorter median overall survival (7.2 months versus 17.5 months; *p* = 0.083, data not shown). However, a prognostic value was obtained when considering the neutrophil count alone. Such an association between blood baseline neutrophil count and Bv efficacy in GBM has previously been reported, but with contradictory results as compared with ours. In the retrospective cohort reported in the study of Bertaut et al. [27], GBM patients were treated mostly with radio-chemotherapy as a first-line and 60% of them received a Bv-based regimen at recurrence. Neutrophil count was performed before the initiation of the first treatment and overall survival was calculated from the date when therapy started to the date of death. In the whole cohort of patients, a high neutrophil count (> 6 G/L) was associated with poorer survival, but this association remained only in the group of patients not receiving Bv. The authors concluded that Bv was able to counterbalance the deleterious effect of a high neutrophil count. In this study, overall survival was the result of the initial treatment, comprising temozolomide (TMZ) and the treatment at recurrence, with or without Bv. In this case, the status of O^6^-methylguanine DNA methyltransferase (*MGMT*) promoter methylation, which is a very powerful predictor of response to TMZ for newly diagnosed GBM patients, with a PFS difference of approximately 7 months between “methylated” and “unmethylated” patients, [28] should have been taken into account. However, this was not the case and could have induced a bias in the reported result. On the other hand, given the significant fluctuations in neutrophil counts, especially those induced by corticosteroid treatment, it seems preferable to consider the neutrophil count at the beginning of Bv treatment, as was done in our study. In any case, only complementary prospective studies will allow the validation, or not, of these different results.

Another potential interesting biomarker associated with overall survival in our prospective cohort of patients was the basal absolute count of Treg. As Treg contribute to immunosuppressive mechanisms, high levels of these cells are intuitively expected to correlate with a poor prognosis. However, if a high tumour infiltration by Treg is significantly associated with shorter overall survival in many solid tumours, the opposite can be observed [29–31]. In GBM, in particular, some studies have shown a prognostic value of Foxp3^+^ tumour-infiltrating lymphocytes [32], and others not [33, 34]. In any case, lymphocytes and especially Treg remain a minority contingent of immune cells in GBM tumours compared to myeloid cells. Concerning the prognostic impact of peripheral blood Treg, the same phenomenon can be observed. For example, in patients with follicular lymphoma, high levels prior to therapy have been associated with decreased PFS [35]. On the other hand, no correlation was observed between the pre-treatment percentage of circulating Treg and survival in newly diagnosed GBM patients, [33] whereas in diffuse large B-cell lymphoma (DLBCL), a low number of circulating Treg has been shown to be associated with poor prognosis [36]. The prognostic value of Treg seems to vary according to the type of tumour, but also according to the treatment. We have also to keep in mind that Treg are not a homogeneous population. Different stages of activation/differentiation have been described based on the expression of markers, such as CD127/CD152/CD45RO. In patients with non-small cell lung cancer (NSCLC) treated with front-line chemotherapy, an increased percentage of baseline naive Treg is associated with a poor clinical outcome, whereas high baseline levels of terminal effector Treg is correlated with improved clinical response [37]. As for neutrophils, it would therefore be interesting to check if peripheral Tregs found in GBM patients at recurrence display a particular activation profile. In our prospective cohort of patients exclusively treated at recurrence with Bv, a high level of Treg, defined as CD3^+^CD4^+^CD25^+^FOXP3^+^, was linked to a better prognosis. In contrast to neutrophils, Treg levels and their prognostic significance were not influenced by corticosteroid use. This could be regarded as an advantage for using Treg as a prognostic marker instead of neutrophils. However, we believe that neutrophils respond better to the quality criteria required for a good biomarker, such as robustness, reproducibility and accessibility.

The overall survival observed in our two cohorts of patients treated at recurrence with Bv are consistent with those reported in the literature [38]. However, to our knowledge, no biomarker to date has identified a population with such a long median survival. In the study of Tabouret et al., for example, high plasma matrix metalloproteinase-2 levels in recurrent HGG receiving Bv were associated with a better overall survival of 12.8 months, [39] compared to 41 months for corticosteroid-free patients with a low neutrophil count in our validation cohort. Of course, this difference could be due to the selection bias of patients likely to receive Bv at the phase of relapse. Nevertheless, we believe that these new criteria (neutrophil count and corticosteroid intake) should be tested in the large clinical studies previously reported that have used Bv in GBM at relapse and could easily be entered into the current practice of oncologists.

## Electronic supplementary material

Below is the link to the electronic supplementary material.

Supplementary material 1 (PDF 383 KB)

## Abbreviations

Bv: Bevacizumab
CBC: Complete blood cell count
FCM: Flow cytometry
GBM: Glioblastoma
MDSCs: Myeloid-derived suppressor cells
MGMT: O^6^-methylguanine DNA methyltransferase
M-MDSCs: Monocytic myeloid-derived suppressor cells
NLR: Neutrophil–lymphocyte ratio
NSCLC: Non-small cell lung cancer
TEM: Tie 2 expressing monocytes
TMZ: Temozolomide
Treg: Regulatory T cells
VEGF: Vascular endothelial growth factor
