# Supplementary material for: Absolute numbers of regulatory T cells and neutrophils in corticosteroid-free patients are predictive for response to bevacizumab in recurrent glioblastoma patients
Source: Cancer Immunol Immunother. 2019 Mar 4;68(6):871–82. doi: 10.1007/s00262-019-02317-9 (PMC6529384; doi:10.1007/s00262-019-02317-9)
Supplement: Supplementary file 1 — Supplementary material 1 (PDF 383 KB) [file 262_2019_2317_MOESM1_ESM.pdf]

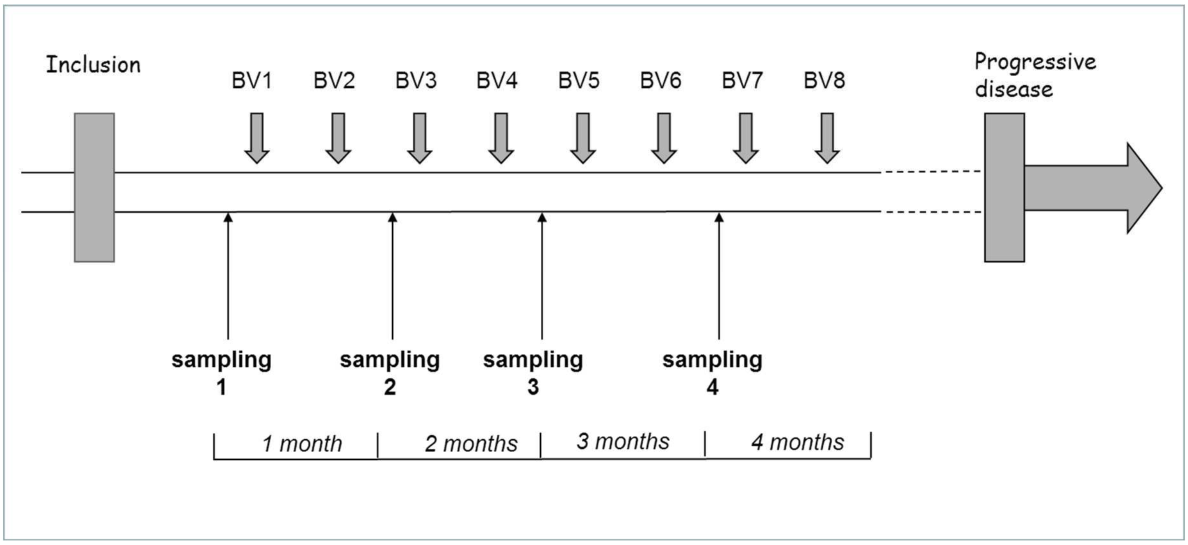

Sup Figure1: **Flow chart showing the study design.**

A/

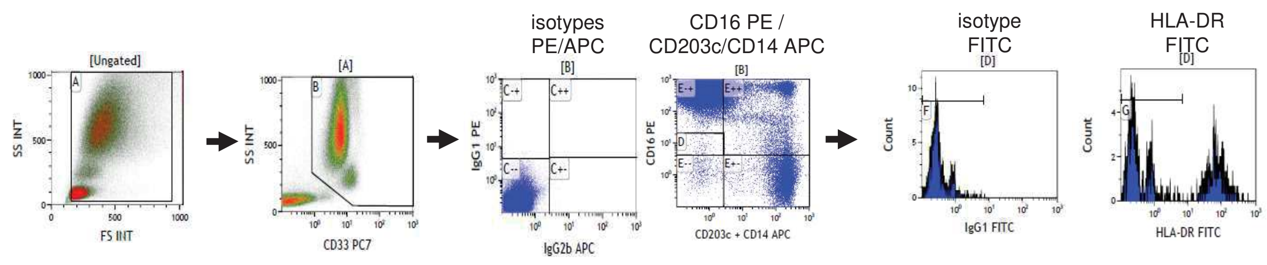
[Ungated]
SS INT
FS INT
[A]
CD33 PC7
isotypes
PE/APC
[B]
IgG1 PE
IgG2b APC
CD16 PE /
CD203c/CD14 APC
CD16 PE
CD203c + CD14 APC
isotype
FITC
[D]
Count
IgG1 FITC
HLA-DR
FITC
HLA-DR FITC

B/

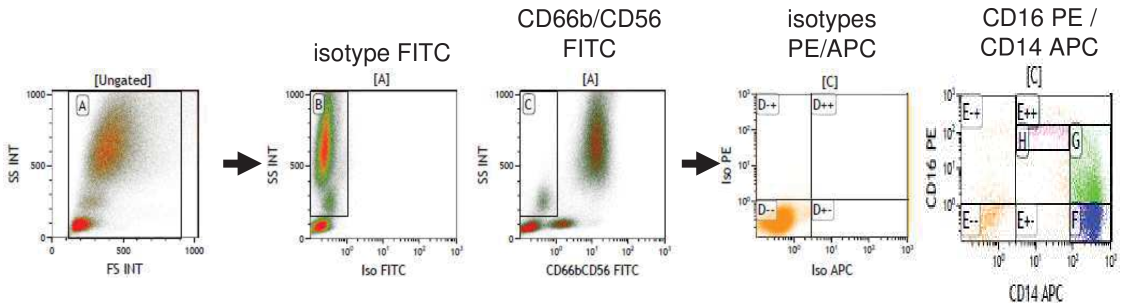
[Ungated]
SS INT
FS INT
isotype FITC
[A]
Iso FITC
CD66b/CD56
FITC
CD66bCD56 FITC
isotypes
PE/APC
[C]
Iso PE
Iso APC
CD16 PE /
CD14 APC
CD16 PE
CD14 APC

C/

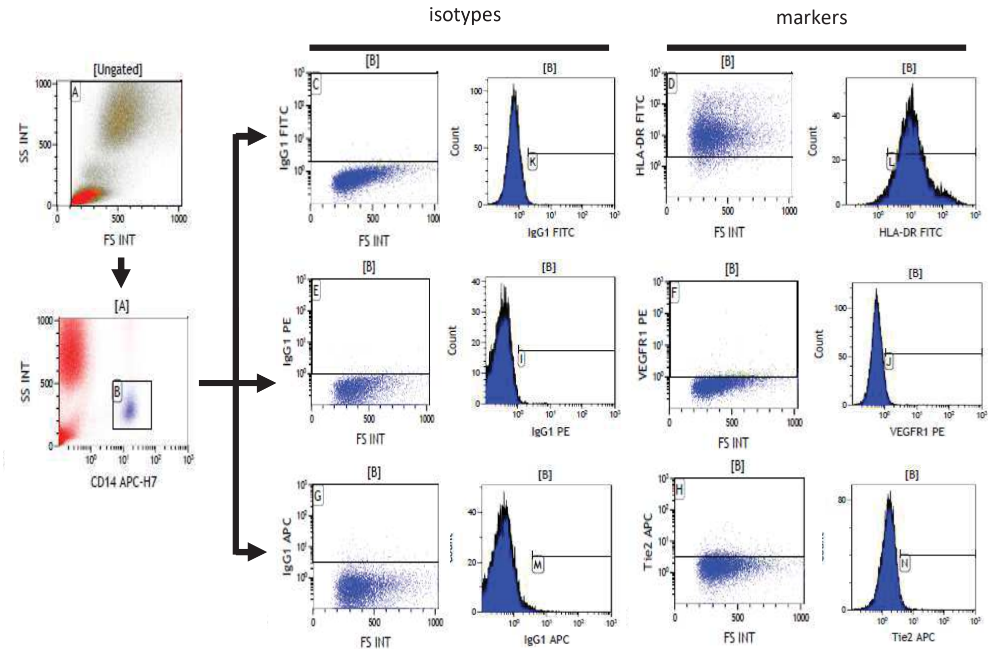
isotypes
markers
[Ungated]
SS INT
FS INT
[A]
CD14 APC-H7
[B]
IgG1 FITC
HLA-DR FITC
IgG1 PE
VEGFR1 PE
IgG1 APC
Tie2 APC
Count

D/

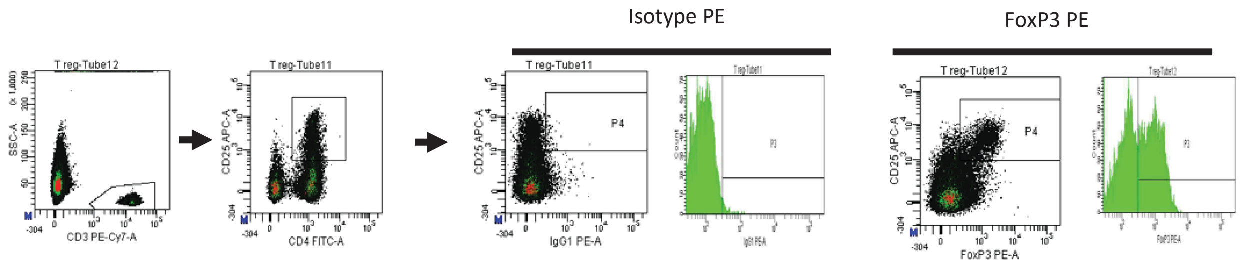
Isotype PE
FoxP3 PE
T reg-Tube12
SSC-A
CD3 PE-Cy7-A
T reg-Tube11
CD25 APC-A
CD4 FITC-A
P4
IgG1 PE-A
FoxP3 PE-A

Sup Figure 2: FACS analysis of the different leukocyte populations. For each tube, 200 µl of whole blood was first incubated with 20 µl of Fc blocking for 5 minutes. A second 20-minute incubation period was performed after the addition of the mAb, followed by red blood cell lysis. Finally, samples were diluted with 0.5 ml PBS before analysis. **A/ MDSCs analysis**. MDSC were defined as $CD33^+/CD203^-/CD14^-/CD16^{-/low}/HLA\text{-}DR^-$. After debris exclusion in a forward/side scatter (FSC/SCC), $CD33^+$ cells were selected and separated based on CD16/CD14 and CD203c expression. Isotype controls were used to define CD16-CD14-CD203c- cell population and HLA-DR positive cells. **B/ Different monocytes subsets**. classical, intermediate and non-classical monocytes were identified as $CD14^{high}/CD16^-$, $CD14^{high}/CD16^+$ and $CD14^{low}/CD16^+$, respectively. After debris exclusion in a forward/side scatter (FSC/SCC), the population $CD56^-$/CD66b- with an intermediate side scatter was selected. Isotype controls were used to define CD66b/CD56 negative cells and to set the quadrant to define CD14/CD16 expression on monocytes. **C/ HLA-DR-, VEGFR1 and Tie2 expression on $CD14^+$ monocytes**. After debris exclusion in a forward/side scatter (FSC/SCC), $CD14^+$ cells were selected and percentages of cells not expressing HLA-DR, expressing VEGFR1 or Tie2 were calculated. Isotype controls were used to define HLA-DR, VEGFR1 and Tie2 positive cells. **F/ Treg**. A step of fixation/permeabilisation of cells was done before intracytoplasmic FOXP3 staining. Treg cells were identified as $CD3^+/CD4^+/CD25^{high}/FOXP3^+$ cells. Isotype controls were used to define FoxP3 positive T cells.

Supplementary Table I. List of antibodies used for FACS analysis

Antibody	Manufacturer	Reference
CD3-PECy7	Beckman Coulter	737657
CD4-FITC	eBiosciences	22-0425-73
CD14-APC	Becton Dickinson	345787
CD14-APCH7	Becton Dickinson	560180
CD16-PE	Becton Dickinson	555407
CD25-APC	eBiosciences	22-0425-73
CD33-PECy7	Beckman Coulter	A54824
CD56-FITC	Becton Dickinson	345811
CD66b-FITC	Beckman Coulter	IM0531U
CD203c	BioLegend	324610
FOXP3-PE	eBiosciences	12-4776-42
HLA-DR-FITC	Beckman Coulter	I1638U
Tie2-APC	R&D	FAB3131A
VEGFR1-PE	R&D	FAB321P
